# Laue microdiffraction on polycrystalline samples above 1500 K achieved with the QMAX-µLaue furnace

**DOI:** 10.1107/S1600576724001821

**Published:** 2024-03-31

**Authors:** Ravi Raj Purohit Purushottam Raj Purohit, Daniel Fowan, Stephan Arnaud, Nils Blanc, Jean-Sébastien Micha, René Guinebretière, Olivier Castelnau

**Affiliations:** a CEA, IRIG, MEM, CNRS, Université Grenoble Alpes, 17 Avenue des Martyrs, Grenoble 38000, France; b IRCER (UMR CNRS 7315), Université de Limoges, 12 Rue Atlantis, Limoges 87068, France; c Institut Néel (UPR CNRS 2940), Université Grenoble Alpes, 25 Avenue des Martyrs, Grenoble 38042, France; d SYMMES (UMR CNRS 5819), Université Grenoble Alpes, CEA, 17 Avenue des Martyrs, Grenoble 38054, France; e PIMM (UMR CNRS 8006), CNRS, ENSAM, Cnam, HESAM, 155 Boulevard de l’Hopital, Paris 75013, France; Ecole National Supérieure des Mines, Saint-Etienne, France

**Keywords:** Laue microdiffraction, high temperatures, phase transitions, microstructure, strain fields, polycrystalline materials, QMAX furnace

## Abstract

X-ray Laue microdiffraction aims to characterize microstructural and mechanical fields in polycrystalline specimens at the sub-micrometre scale with a strain resolution of ∼10^−4^. Here, a new and unique Laue microdiffraction setup and alignment procedure is presented, allowing measurements at temperatures as high as 1500 K, with the objective to extend the technique for the study of crystalline phase transitions and associated strain-field evolution that occur at high temperatures. A method is provided to measure the real temperature encountered by the specimen, which can be critical for precise phase-transition studies, as well as a strategy to calibrate the setup geometry to account for the sample and furnace dilation using a standard α-alumina single crystal. A first application to phase transitions in a polycrystalline specimen of pure zirconia is provided as an illustrative example.

## Abstract 2

A unique X-ray Laue microdiffraction setup and alignment procedure allowing measurements of the microstructure and strain field in polycrystalline specimens at temperatures as high as 1500 K has been developed at beamline BM32, ESRF, making use of a new version of the QMAX furnace.

## 1. Introduction

Laue microdiffraction is a specialized scattering technique available at select synchrotron beamlines, such as 34-ID-E at APS, 12.3.2 at ALS and BM32 at ESRF, where this study was conducted. The technique is devoted to local analysis of the microstructure of polycrystalline specimen surfaces, with a sub-micrometric spatial resolution. Using Laue microdiffraction, one can obtain 2D or even 3D maps that reveal details about crystal structure, crystal orientation and lattice strain (Tamura *et al.*, 2002 ▸; Chung & Ice, 1999 ▸). The technique is based on scanning of the specimen surface with a highly focused polychromatic X-ray beam in reflection mode. This scan produces a Laue diffraction pattern at each beam position, captured by the 2D detector positioned close (a few centimetres) to the specimen surface. The technique has proven invaluable for various applications, including phase identification of tiny crystals (Dejoie *et al.*, 2014 ▸), phase transformation at multi-scales (Guo *et al.*, 2011 ▸; Mun *et al.*, 2011 ▸), crystal orientation mapping (Ma *et al.*, 2015 ▸), elastic strain/stress measurement (Chen *et al.*, 2015 ▸; Kunz *et al.*, 2009 ▸), defect quantification (Ohashi *et al.*, 2009 ▸; Qian *et al.*, 2016 ▸; Lupinacci *et al.*, 2015 ▸) and identification of constitutive relations (Plancher *et al.*, 2017 ▸).

The ‘standard’ Laue microdiffraction technique enables a relatively quick analysis of the specimen with modern detectors (∼10 images s^−1^), allowing an evaluation of the crystal orientation field with a very high accuracy (∼0.005°) and determination of the five components of the deviatoric elastic strain field with an accuracy of ∼10^−4^. Measuring the crystal unit-cell volume requires additional efforts, but this is possible thanks to the rainbow method (Robach *et al.*, 2013 ▸) or by inserting a monochromator in the incoming beam path (Ice *et al.*, 2000 ▸). The measurement of the complete (not only deviatoric) local elastic strain tensor is a necessary step for estimating the associated stress when the crystal structure departs from a cubic one [see the supporting information of Petit *et al.* (2015 ▸) for further details]. Depth-resolved analyses with micrometre resolution are also possible due to the differential-aperture X-ray microscopy technique (Larson *et al.*, 2002 ▸; Marijon, 2017 ▸; Hektor *et al.*, 2018 ▸), which consists of shadowing in successive steps part of the diffracted Laue pattern with a small absorbing wire.

Once data are collected, quantitative analysis relies on comparing computed Laue pattern images with measured ones, while matching the orientation of the diffracting crystal and its unit-cell parameters in order to minimize the difference between the two. Advanced digital image-correlation techniques further improve the accuracy of crystal orientation and lattice strain measurements by analysing the relative distortion between two Laue patterns (*e.g.* during *in situ* mechanical tests), making the results less sensitive to inaccuracies related to the setup calibration (Petit *et al.*, 2015 ▸; Zhang *et al.*, 2015 ▸, 2017 ▸). This can be used for example to identify the material constitutive relation at the sub-micrometre scale (Plancher *et al.*, 2017 ▸, 2019 ▸). Several open-source software tools have been developed at synchrotron beamlines and are available to users. Treatment of the tremendous volume of data has become quite cumbersome with the speedup of image acquisition and the increase in image size. Recently, machine-learning approaches, such as neural networks, have enabled efficient indexing methods for large datasets by attributing the correct *hkl* indices to each Laue spot of the pattern (Purushottam Raj Purohit *et al.*, 2022*a* ▸). This allows data analysis to be performed almost synchronously with data acquisition, even for low-symmetry crystal structures. On the other hand, quantitative data analysis relies on an accurate calibration of the used setup, *i.e.* the position and orientation of the X-ray detector with respect to the incoming beam must be known to a high accuracy, and the sample surface must be positioned precisely at the diffraction centre. At BM32, this is achieved with the use of an optical microscope with a depth of field of ∼1 µm.

Despite these advancements, most existing techniques and applications have operated so far at room temperature. Only a limited number of studies have explored different temperature ranges. For example, higher temperature (up to 873 K) was used *in situ* by Schneider *et al.* (2012 ▸) for the study of phase transitions in (GeTe)_*n*_Sb_2_Te_3_ using a domed hot stage. In this study, the sample position was adjusted to account for the stage dilation, but the accuracy with which the unit-cell parameters could be extracted was limited to 0.1 or 1%, which is not enough for elastic strain (and related residual stress) analyses. The phase transition in Fe–Cr alloys has been studied by Al Khoury *et al.* (2018 ▸), but at room temperature in quenched specimens. Investigations of detwinning in electrodeposited Cu have been carried out at low temperature (120 K) using a setup where the specimen was cooled with a liquid-nitro­gen gun (Wang *et al.*, 2010 ▸).

Extending the capabilities of Laue microdiffraction to very high temperatures is a critical advancement for various scientific and industrial applications. High-temperature conditions are pivotal for understanding the behaviour of materials in extreme environments, such as those found in aerospace engines, nuclear reactors and manufacturing processes involving high-temperature phase transitions. As materials are subjected to high temperatures, their crystalline structure undergoes transformations, and new phases can emerge. The ability to accurately characterize these changes with Laue microdiffraction would offer unprecedented insights into the thermodynamic and kinetic processes governing material behaviours at high temperatures.

In this study, our objective is to expand the range of Laue microdiffraction to accommodate extremely high temperatures, targeting up to 1500 K in our initial application. Achieving this means overcoming several technical hurdles. First, the equipment setup holding the specimen expands or contracts with temperature changes, requiring recalibration at each temperature level. However, standard calibration procedures are unsuitable for such conditions, primarily because they rely on the use of an optical microscope. This becomes impractical at elevated temperatures, where the specimen must be enclosed within a hemispherical dome. Secondly, a common challenge in all high-temperature experiments is accurately determining the specimen’s real temperature, which may deviate from measurements provided by conventional means like thermocouples. To address these issues, we introduce a novel methodology that allows for accurate calibration and temperature measurement under these extreme conditions.

This article is organized as follows. Section 2 elaborates on the geometry and capabilities of a new version of the QMAX furnace, a furnace specially adapted for high-temperature Laue microdiffraction experiments at beamline BM32. Section 3 details the setup calibration procedures optimized for high-temperature measurements. This also includes an efficient way to directly measure the specimen temperature which can differ from the target temperature. Section 4 describes the first application on a zirconia polycrystalline specimen undergoing a high-temperature monoclinic–tetragonal martensitic phase transition.

## 2. The QMAX-µLaue furnace for Laue microdiffraction

**Figure 1:**
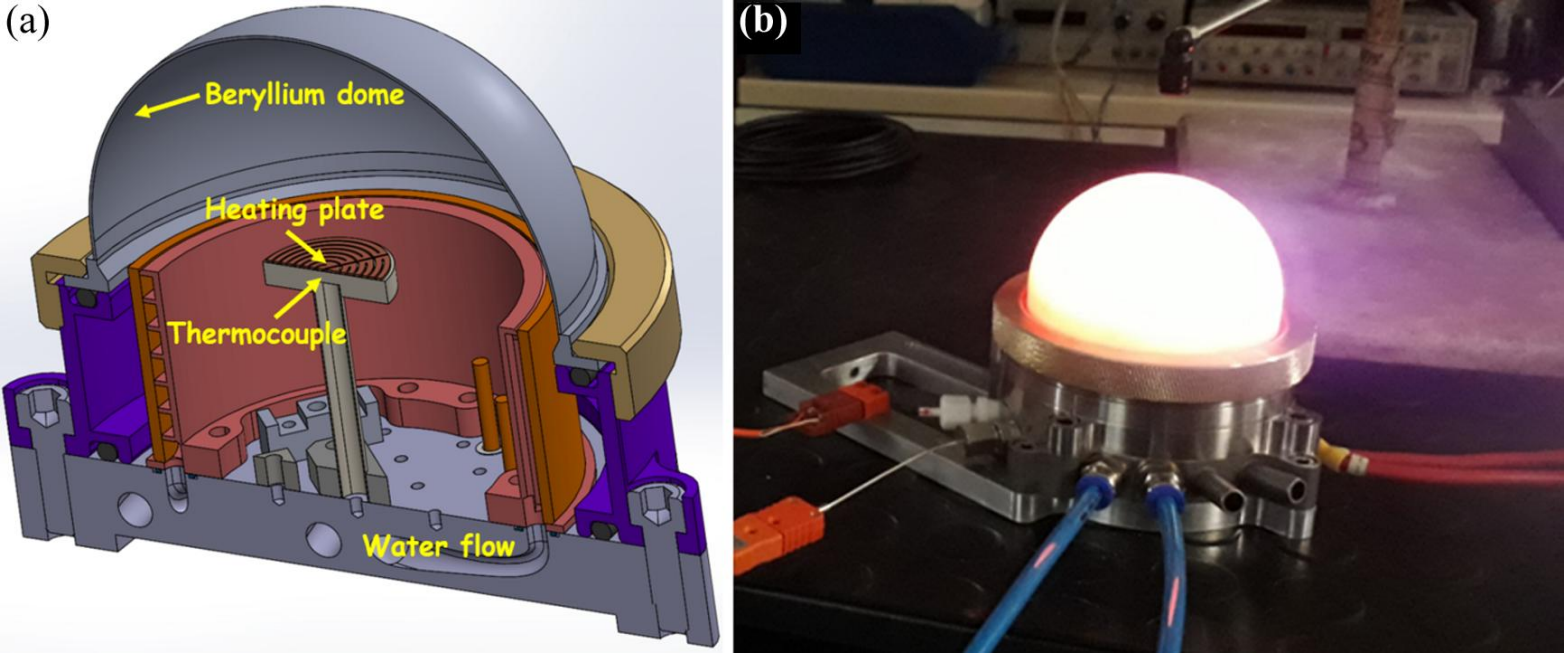
The QMAX-µLaue furnace. (*a*) A 3D drawing of the furnace. (*b*) A photograph of the furnace at 1500 K with the PEEK dome.

The QMAX-µLaue furnace, depicted in Fig. 1 ▸, is a specialized innovation designed to offer unrestricted access to the half-space above the sample. This design feature facilitates the recording of any X-ray beam diffracted in reflection mode. One of the new engineering feats achieved with the QMAX-µLaue furnace, compared with its predecessor (QMAX) used at ESRF’s BM02 beamline for monochromatic X-ray measurements (Guinebretière *et al.*, 2020 ▸), was the considerable size reduction without compromising the ability to record high-temperature diffraction patterns with a 2D X-ray detector positioned at ∼8 cm from the hot samples. The furnace base was redesigned and built using additive manufacturing techniques. The resistor (25 mm diameter) is composed of a 80Pt–20Rh plate mounted on a flat alumina-based ceramic holder. Temperature regulation within the furnace is managed by a Nanodac controller from Eurotherm by Schneider Electric. It works in tandem with a Pt–10% Rh–Pt thermocouple, positioned ∼1 mm below the sample in a small hole (not shown in Fig. 1 ▸) drilled in the ceramic holder below the resistor and parallel to it. These features allow for computer-controlled adjustments of the proportional-integral-derivative parameters of the furnace as a function of temperature, with a maximum heating rate of 50 K min^−1^.

To attain extremely high temperatures, the sample’s surrounding atmosphere must be confined within a hemispherical dome. Moreover, such a dome allows high-temperature measurements under reactive or inert gas atmosphere at the atmospheric pressure, by controlling the input gas flow (Guinebretière *et al.*, 2020 ▸). Two domes are currently available. One is made of polycrystalline beryllium (thickness: 0.5 mm), which is nearly transparent in the X-ray energy range used but creates thousands of tiny parasitic Laue spots on the detector. The second is a polymer dome made of polyether ether ketone (PEEK, thickness: 0.25 mm), which causes some scattering of the incoming and diffracted X-rays but avoids the issue of parasitic Laue spots.

## 3. Experimental procedure

This section briefly revisits the Laue microdiffraction setup available at the BM32 beamline and elaborates on the standard room-temperature calibration procedure. A precise calibration of the setup is important for Laue microdiffraction as lattice strains due to internal stresses in the specimen lead to deviation of the diffracted beam direction of the order of hundredths of a degree. Laue spots should thus be located on the detector screen with a subpixel resolution (*i.e.* tens of micrometres) for accurate assessment of strain components or crystallographic lattice parameters. At room temperature, such an accuracy is reached by making use of an optical microscope for sample alignment (by reflected visible light) and a strain-free Ge single-crystal wafer (Ulrich *et al.*, 2011 ▸; Poshadel *et al.*, 2012 ▸; Robach *et al.*, 2014 ▸; Zhang *et al.*, 2017 ▸). However, this is no longer possible at high temperatures due to the necessary use of a Be or PEEK dome for heat confinement. In the absence of the microscope, we exploit the diffraction pattern of a strain-free sapphire standard whose unit-cell parameter evolution is well known as a function of temperature. We describe the proposed strategy below.

### 3.1. Laue microdiffraction setup and standard calibration procedure

**Figure 2:**
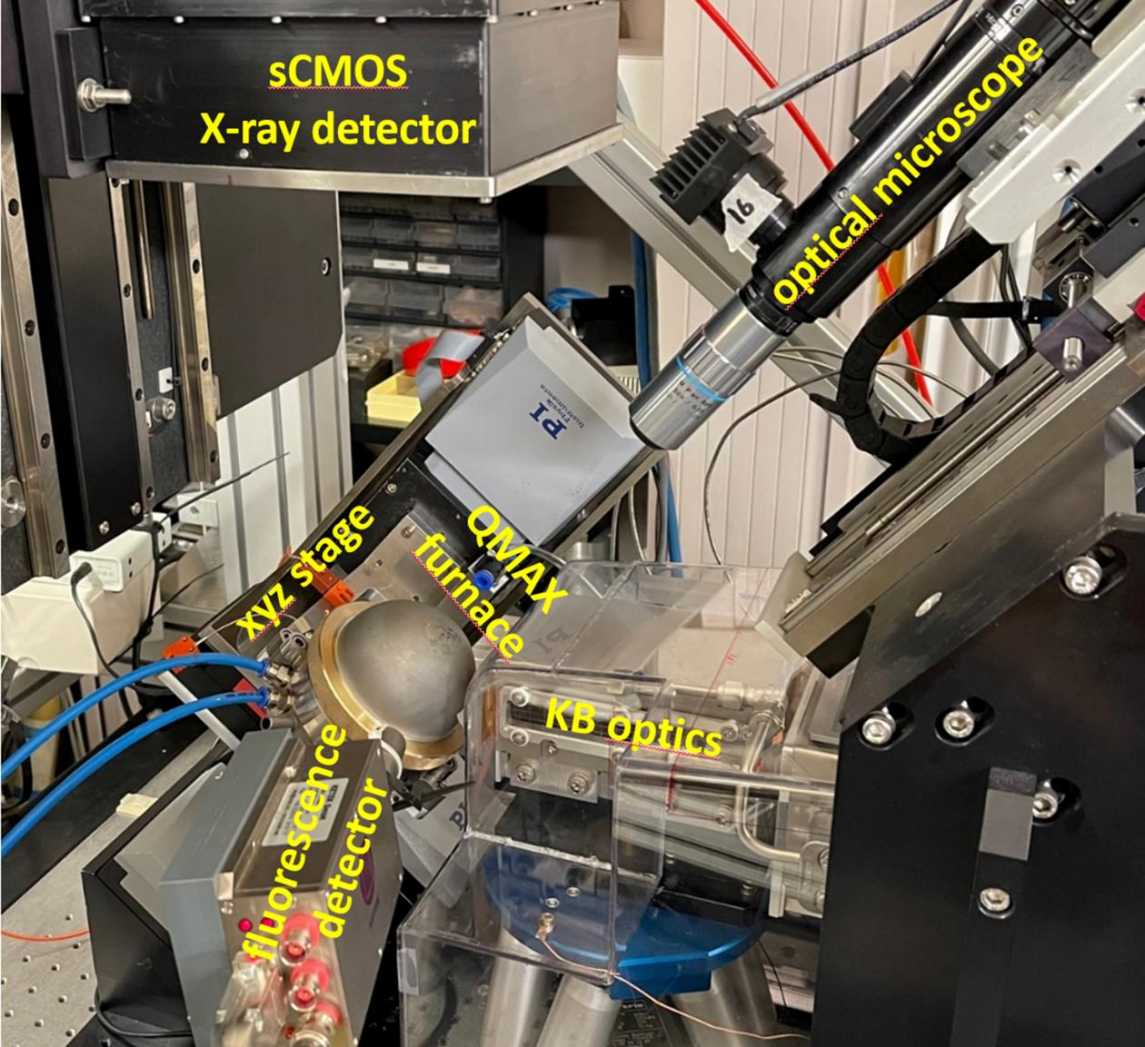
The Laue microdiffraction setup at the BM32 beamline, with the QMAX-µLaue furnace mounted with its Be dome. In this image, the sCMOS detector and the optical microscope have been moved away from the specimen.

The BM32 Laue microdiffraction setup is illustrated in Fig. 2 ▸. It features:

(*a*) A Kirkpatrick–Baez (KB) mirror that focuses the incoming polychromatic beam (energy range 5–23 keV) down to ∼300 × 300 nm.

(*b*) A sample stage with a *xyz*-translation stage for precise sample positioning.

(*c*) An sCMOS 2D detector with a pixel resolution of 4032 × 4036, 16-bit depth and a pixel size of 36.7 µm, situated ∼70 mm above the incoming X-ray beam. The detector surface is approximately parallel to the incoming beam, while the incidence angle of the incoming beam on the specimen surface is ∼40°.

(*d*) An optical microscope with adjustable working distances of 13 or 35 mm, used for sample positioning and region of interest (ROI) alignment.

(*e*) An optional fluorescence detector for defining the ROI when surface features are invisible. This detector provides valuable data on the distribution of chemical elements within the sample.

**Figure 3:**
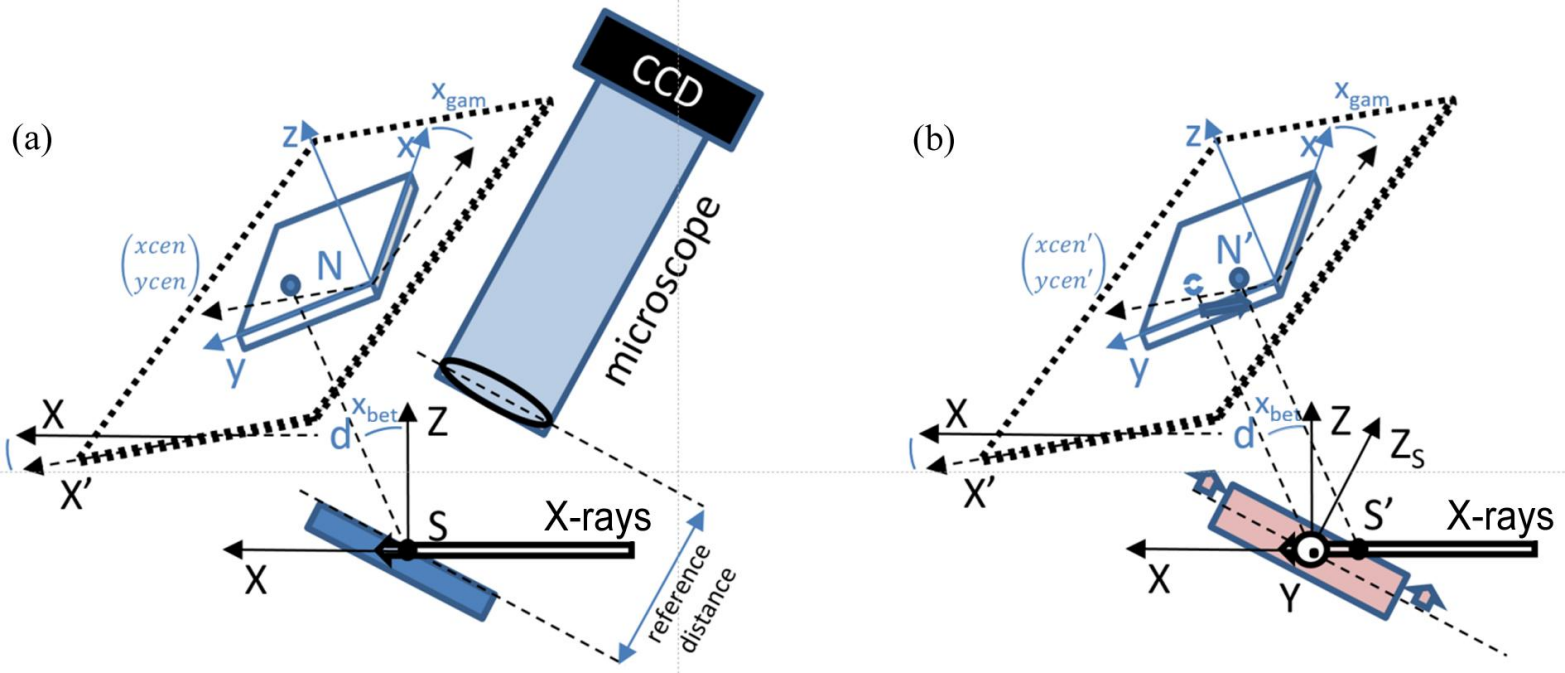
(*a*) A schematic drawing of the Laue microdiffraction detection geometry at BM32. Five parameters describe the relative position and orientation of the detector with respect to the incoming X-ray beam direction and a point at the sample surface S of the pixelated array (blue). *x*_bet_ and *x*_gam_ are two tilt angles usually close to 0°. The distance *d* separates S from the nearest point N on the detector plane (black), whose coordinates in pixel units are xcen and ycen. For the calibration and the specimen measurements, the sample surface (tilted by ∼40° from the horizontal plane) can be positioned accurately with respect to the microscope objective at a constant reference distance, ensuring the analytical computation of scattering angles of Laue spots coming from S and hitting the detector. (*b*) The same schematic drawing in the case of an *in situ* experiment without an optical microscope and with thermal drift of the sample surface. Without the microscope and due to the vertical thermal dilation of the sample along Z_S_ (red arrows), Laue spots originate from S′ and new calibration parameters must be used. If *x*_bet_ = *x*_gam_ ≃ 0 and the sample surface does not rotate significantly, only the value of ycen is modified (blue arrow, see main text).

A more comprehensive description of the setup at BM32 is detailed by Ulrich *et al.* (2011 ▸). At room temperature, the standard procedures for calibration of the setup and measurement of the specimen run as follows (Robach *et al.*, 2014 ▸):

(1) Microscope alignment: align the optical microscope with the incoming beam using thin perpendicular lines (2 µm wide and 0.25 µm thick) of Cu or Au deposited on a substrate at the sample position. As the lines can be observed with both the X-rays (fluorescence emitted signal) and the optical microscope, and since the depth of view of the microscope is very small, this enables precise localization of the incoming X-ray beam in relation to the optical microscope.

(2) 2D detector positioning: utilize a stress-free Ge single-crystal wafer with well known unit-cell parameters to determine the 2D X-ray detector’s position. Following step (1) above, the surface of the single crystal is positioned right into the incoming beam and in the focal plane of the optical microscope, as illustrated in Fig. 3 ▸(*a*). The Laue pattern recorded on the detector contains Laue spots whose positions are given by the crystal orientation and detector position and orientation. Five calibration parameters describe the detector pixel array geometry: two angles giving the orientation of the detector surface with respect to the incoming beam and three distances (detector-plane-to-sample distance and two pixel coordinates of the nearest point of the detector to the sample). Dedicated software, *LaueTools*, available at https://github.com/BM32ESRF/lauetools, is used for identifying the five calibration parameters [denoted as *x*_gam_, *x*_bet_, xcen, ycen and *d*; refer to Fig. 3 ▸(*a*)] by minimizing the distance between the predicted and measured Laue spot positions on the detector screen for the stress-free Ge crystal.

(3) Specimen measurements: replace the calibration single crystal with the specimen to be studied, positioning it on the *xyz* stage with its surface lying within the microscope focal plane. Utilizing the previously determined calibration parameters, Laue pattern data can be analysed to ascertain lattice parameters and crystal orientation.

The absence of precise Laue spot energy measurements constrains the data to five deviatoric components of the strain tensor or the shape description of the unit cell: *b*/*a*, *c*/*a*, α, β and γ. Strain resolution by Laue microdiffraction, using the standard procedure for circular and sharp Laue spots, is typically of the order of 10^−4^ (Poshadel *et al.*, 2012 ▸; Petit *et al.*, 2015 ▸; Zhang *et al.*, 2017 ▸).

### 3.2. Setup calibration at high temperatures, and temperature measurement

#### 3.2.1. High-temperature calibration challenges

At high temperatures, due to the enclosure within the dome, we are in a configuration where the microscope cannot be used, and the X-ray beam’s ROI is probably different from the initial setting at room temperature, that is, the specimen surface is probably beyond the focal plane of the microscope [Fig. 3 ▸(*b*)]. This changes the part of the sample probed by the X-ray beam, *i.e.* the incoming beam is no longer scanning exactly the same ROI as at room temperature. On the X-ray detector plane, apart from the Laue pattern distortion due to specimen dilation, the displacement of the sample surface away from the focal plane leads to a translation of all Laue spots (Robach *et al.*, 2014 ▸; Petit *et al.*, 2015 ▸) by a distance of about SS′ and in a direction parallel to the incoming X-ray beam, which can be estimated for each temperature. This should correspond to variations in the xcen, ycen calibration parameters (the centre of recorded diffraction patterns), while the other parameters (*x*_gam_, *y*_bet_ and *d*) are expected to remain consistent with their room-temperature values.

Determining the actual temperature of the specimen during high-temperature experiments presents another challenge rarely considered in the literature. Factors such as thermocouple accuracy, internal furnace temperature gradients, sample thickness and the thermocouple’s position relative (a few millimetres away) to the ROI can cause discrepancies between the set-point and actual sample temperatures.

#### 3.2.2. Proposed procedure

To simultaneously calibrate the Laue setup and ascertain the true specimen temperature, we adapt a methodology involving the detailed analysis of diffraction patterns from a single crystal of stress-free sapphire placed adjacent to the sample under study. Sapphire is selected due to its well documented temperature-dependent lattice parameters *a* and *c* (Touloukian *et al.*, 1977 ▸). We employ a flat piece of stress-free sapphire, of similar thickness to the studied specimen, and cut parallel to the (006) plane family. A single Laue diffraction pattern from this sapphire contains a large number of diffraction spots (∼160 spots on a detector placed 79.5 mm from the sample in reflection for an energy range of 5–22 keV), the positions of which directly inform us about the cell parameters. These cell parameters are both temperature and strain dependent. For a stress-free α-alumina single crystal and according to the hexagonal setting of the 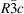 space group, they evolve following the polynomial laws below (expressed in percentages), where the temperature *T* is given in kelvin (Touloukian *et al.*, 1977 ▸):

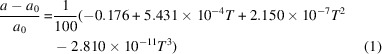

and

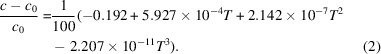

The sapphire crystal and the specimen are both intentionally kept small, typically around 5 mm, in comparison to the 25 mm diameter Pt–Rh resistor. This size selection is important for minimizing lateral temperature gradients in the specimen, and their proximity further aids in reducing temperature discrepancies between them. To ensure uniformity in calibration, their thicknesses are similarly kept under 1 mm. Additionally, any surface elevation differences between the sapphire and the specimen measured at room temperature are accounted for in the high-temperature calibration corrections. Here, we have also adopted a gradual heating approach with a small rate of ∼20 K min^−1^, temperature increments of a few tens of degrees and a five-minute stabilization period before initiating Laue scans, which last around 45 min each. These measures – along with the similar geometries of the sample and sapphire, slow heating/cooling rates, and extended temperature plateaus – lead us to anticipate that the temperatures of the sapphire crystal and the specimen will be closely matched. The temperature measurements represent a volume average across a significant portion of the sample thickness, facilitated by the penetration depth of the incoming X-rays which is of the same order as the specimen thickness (at 17 keV, the X-ray attenuation length is 650 µm for Al_2_O_3_ and 160 µm for ZrO_2_).

To accurately identify the real temperature of the sapphire crystal on the basis of its diffraction pattern, we propose the iterative calibration scheme described below. This also allows us to extract a set of calibration parameters for the setup at any given temperature. Initially, a room-temperature calibration is conducted to establish the five baseline calibration parameters, using the known cell parameters of sapphire at room temperature.

*Initial setup*. Given a set-point temperature *T*_0_, we calculate the stress-free lattice parameters of sapphire (*a*_*T*0_, *c*_*T*0_) using polynomial laws (1) and (2).

Using the room-temperature calibration parameters as an initial starting point, we perform a stepwise refinement of the calibration parameters. Specifically, we first adjust the coordinates (xcen, ycen) of point N, followed by the sample–detector distance *d*, and finally the detector tilt angles, all through least-squares optimization.

*Iterative calibration*. The iterative procedure of geometry calibration is carried out as follows:

Step 1: during each *i*th iteration, we optimize only the *c* lattice parameter using least-squares minimization. From this, we derive a new temperature *T_i_*, using equation (2).

Step 2: utilizing the newly estimated temperature *T_i_*, we extract a new set of lattice parameters (*a_Ti_*, *c_Ti_*) based on polynomial laws (1) and (2).

Steps 1 and 2 are continually repeated, updating the temperature *T_i_* in each cycle. Each new iteration begins with the cell parameters estimated in the previous iteration (*i* − 1). The iterative process terminates when the lattice parameters stabilize and are consistent with the set-point temperature. The procedure works identically well when optimizing the lattice parameter *a* in Step 1 instead of *c* and estimating *c* with respect to the new temperature using equation (2).

**Figure 4:**
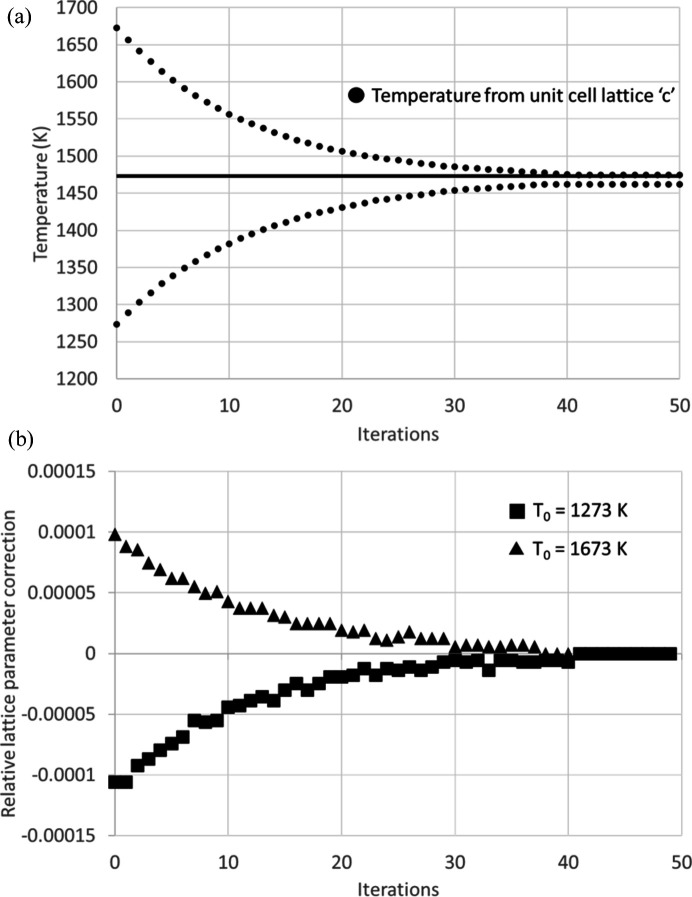
Convergence of the proposed iterative scheme for a synthetic Laue pattern of a sapphire single crystal. (*a*) Temperature derived at each iteration from the refined *c* and calibration parameters. Two different initial guesses as the starting point (1673 K, 1273 K) are shown. The true temperature is 1473 K. (*b*) Relative lattice parameter correction as a function of the iteration step for the two different initial *T*_0_.

This procedure has been validated using both synthetic and real data. In Fig. 4 ▸(*a*), we illustrate the convergence of this method using simulated Laue patterns of sapphire (synthetic data) for a temperature of 1473 K, with the typical setup geometry employed at BM32. To test the robustness against different initial conditions, we started with two different set-point temperatures far from 1473 K (*T*_0_ = 1673 K and *T*_0_ = 1273 K). In both cases, the scheme converged to the accurate temperature within ∼50 steps, achieving an accuracy better than 10 K.

Fig. 4 ▸(*b*) reports the relative lattice parameter mismatch correction, calculated as (2/3)[(*c*_*i*_ − *c*_*i*−1_)/*c*_*i*−1_], at the end of each iteration. This mismatch continually decreases in a monotonic manner, eventually reaching an accuracy better than 10^−5^.

**Figure 5:**
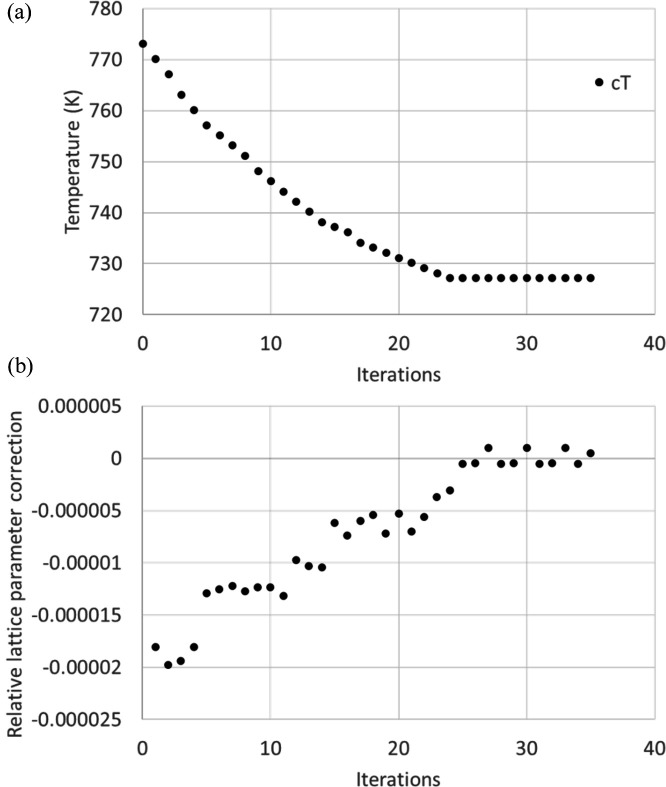
Convergence of the proposed iterative scheme using a measured Laue pattern in sapphire. (*a*) The iterative convergence process of temperature obtained when the *c* lattice parameter is made free, starting with an initial guess of 773 K. (*b*) Correction of the relative cell parameters at each step of the iterative convergence process.

We report now the behaviour of the above iterative procedure on experimental data measured on sapphire (001). Fig. 5 ▸ illustrates the obtained convergence rate for a Laue pattern recorded for a set-point temperature of 773 K. The procedure converges quickly, in ∼25 steps, a consequence of a good initial guess. The final temperature obtained is 727 K. The lattice parameter converges with an accuracy better than 10^−5^, similar to the synthetic data, which shows that the proposed method is accurate enough for the study of lattice strain and residual stresses at high temperature.

**Figure 6:**
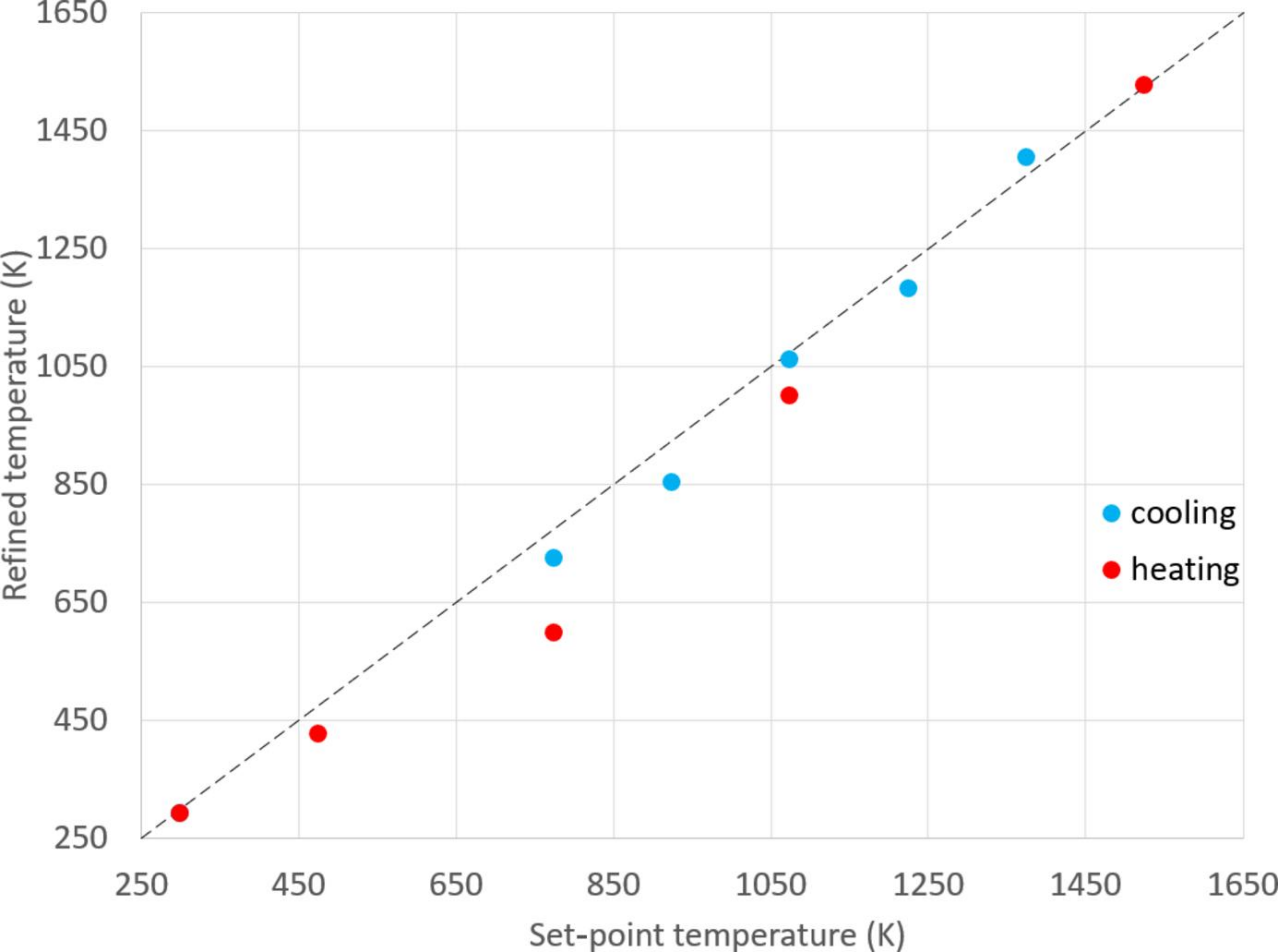
Refined temperature resulting from the iterative calibration process as a function of the set-point temperature during heating (red) and cooling (blue), up to 1500 K.

The same method has been employed to determine the true temperature during an actual experiment in which the QMAX-µLaue furnace was used to study a ZrO_2_ specimen up to 1500 K (see Section 4). Fig. 6 ▸ plots the estimated temperatures against their respective set-point temperatures. The findings indicate that the estimated temperatures are on average slightly lower than the set-point temperatures, by 37 K on average across the tested temperature range. This discrepancy appears to be smaller during the cooling phase (22 K) compared with the heating phase (57 K) in this experiment. This shows a systematic gap between the target temperature and the real sample temperature, which is expected but often not considered in published papers. The standard deviation of the refined temperature, compared with the target one, is 52 K, which indicates the typical temperature discrepancy over the whole temperature range considered. These observations are in line with the results of Guinebretière *et al.* (2020 ▸), who found a similar difference of less than 60 K when using a comparable furnace and a monochromatic X-ray diffraction procedure. One reason for this discrepancy is related to the reduced reliability of the Pt–Rh/Pt thermocouples at lower temperatures. Another reason is simply that the temperature of the metal ball that ends the thermocouple is not the same as the temperature inside the oxide sample or inside the piece of sapphire. Henceforth, all temperatures cited in this article will refer to these refined estimates rather than the initial set-point temperatures.

**Figure 7:**
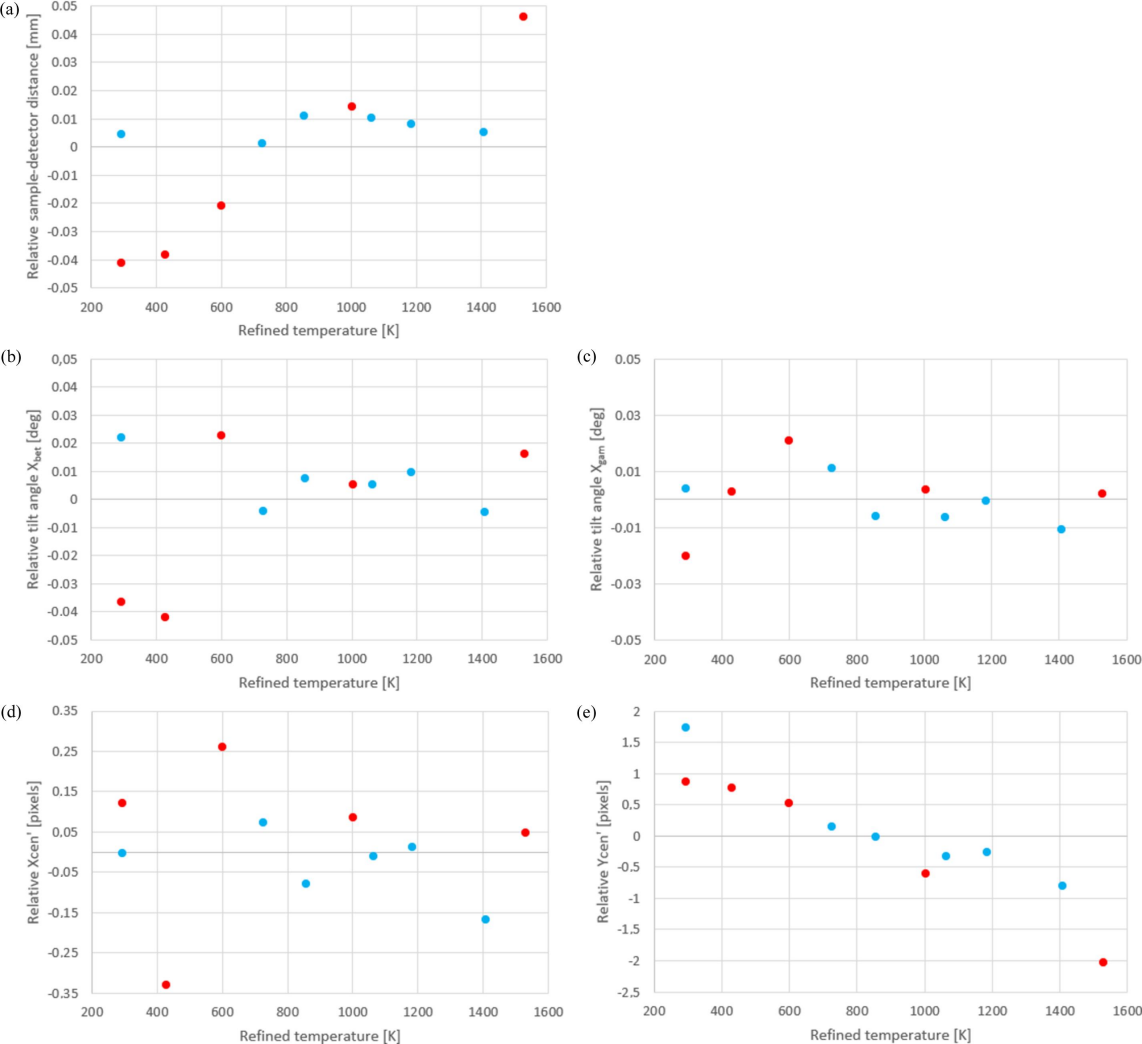
Relative values (with respect to their mean) of the refined detector parameters as a function of refined temperature. (*a*) Sample–detector distance *d*, (*b*) detector tilt angle *x*_bet_, (*c*) detector tilt angle *x*_gam_, (*d*) coordinate xcen of point N and (*e*) coordinate ycen of point N. As in Fig. 6 ▸, the red points were recorded during heating while the blue ones were obtained during the cooling process.

Finally, Fig. 7 ▸ presents the fluctuation (with respect to their mean value) of the optimized calibration parameters for each temperature, as determined through the iterative process described earlier. Among the five calibration parameters, most display negligible changes with temperature. For instance, the standard deviations for *x*_bet_, *x*_gam_ and xcen are 0.02°, 0.01° and 0.15 pixels, respectively. However, the ycen coordinate, which is approximately parallel to the incoming X-ray beam, shows a linear change of approximately four pixels (*i.e.* ∼150 µm) with temperature fluctuations. This variation is attributed to sample and furnace dilation, which in turn affects the volumes probed by the X-ray beam within the single crystal. This leads to alterations in the centre of the Laue diffraction diagram. It is not expected that the calibration parameter ycen be exactly the same during heating and cooling since, due to the very high temperature range considered, the furnace response may not be reversible, as often observed for high-temperature furnaces. These results affirm that the sapphire crystal can be effectively used to calibrate the setup with a high degree of accuracy, even in the absence of an optical microscope. In the first application presented in the following section, these refined calibration parameters are used at each temperature to infer the evolution of the specimen microstructure.

## 4. Application: local cell parameter fluctuation in a tetragonal pure zirconia polycrystal at 1500 K

Using the methodology outlined earlier, good estimates for both the specimen’s true temperature and the calibration parameters of the setup can be determined at each temperature. Here, we showcase the first application of this new setup: a comprehensive examination of a polycrystalline pure zirconia sample.

Zirconia is a very well known oxide used in numerous applications and is renowned for its dual benefits: excellent thermomechanical properties and high ionic conductivity (Kisi, 1998 ▸). Due to the ability of zirconium to accommodate different atomic coordinations, pure zirconia (ZrO_2_) crystallizes in different phases depending on pressure and temperature. At atmospheric pressure, pure unstressed zirconia solidifies into a cubic crystal structure (*c* – space group *Fm* 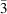 *m*) at ∼2973 K, transforms into a tetragonal structure (*t* – space group *P*4_2_/*nmc*) when cooled to 2573 K and transforms into a monoclinic structure (*m* – space group *P*2_1_/*c*) at 1443 K (Smirnov *et al.*, 2003 ▸). The last transformation, *t → m*, is accompanied by a shear strain and a large anisotropic volume expansion (∼4%). The dilation coefficients of the monoclinic phase are also strongly anisotropic (difference by a factor of about four depending on the considered direction) [see, for example, Guinebretière *et al.* (2022 ▸)]. Therefore, huge residual stresses in the gigapascal range have been shown at room temperature in specimens having encountered the *t → m* transformation (Ors *et al.*, 2021 ▸), and a dense network of microcracks is formed during thermal loading. Guinebretière *et al.* (2022 ▸) showed that the martensitic *t → m* transformation is in fact a continuous one as it spreads over more than 1000 K during cooling. The volume-fraction evolution of the tetragonal phase is well modelled by a sigmoidal law, except in a narrow temperature range close to 1225 K, where intense microcrack formation is expected. A small volume fraction of the high-temperature *t* phase is still present at room temperature, more than 1000 K below the theoretical transition temperature. This can be explained by the combined effects of crystal size and internal stresses. Further evidence of huge elastic strain has been obtained recently using high-temperature 3D mapping of the reciprocal space close to the 111 tetragonal reciprocal lattice nodes (RLNs), around which diffuse scattering stems linked to the 111 and 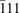 monoclinic RLNs have been observed (Purushottam Raj Purohit *et al.*, 2022*b* ▸). Therefore, it is expected that the huge internal stress field and the associated stress relaxation due to microcracking play an important role during *t → m* phase transformation in such pure zirconia polycrystals. Our high-temperature Laue microdiffraction investigation aims to image local stress-field fluctuations at the sub-micrometre level.

We show here the first results obtained on a polycrystalline specimen of pure ZrO_2_ that was obtained by fuse casting by Saint-Gobain. The manufacturing procedure and microstructure details are detailed by Patapy *et al.* (2013 ▸). During the *in situ* experiments, the specimen was heated from room temperature to 1530 K and then cooled back to room temperature, in many steps (∼50), with varying temperature increments at each step. For instance, during the cooling process, temperature drops as small as 10 K were used to closely observe phase transitions around 1225 K.

**Figure 8:**
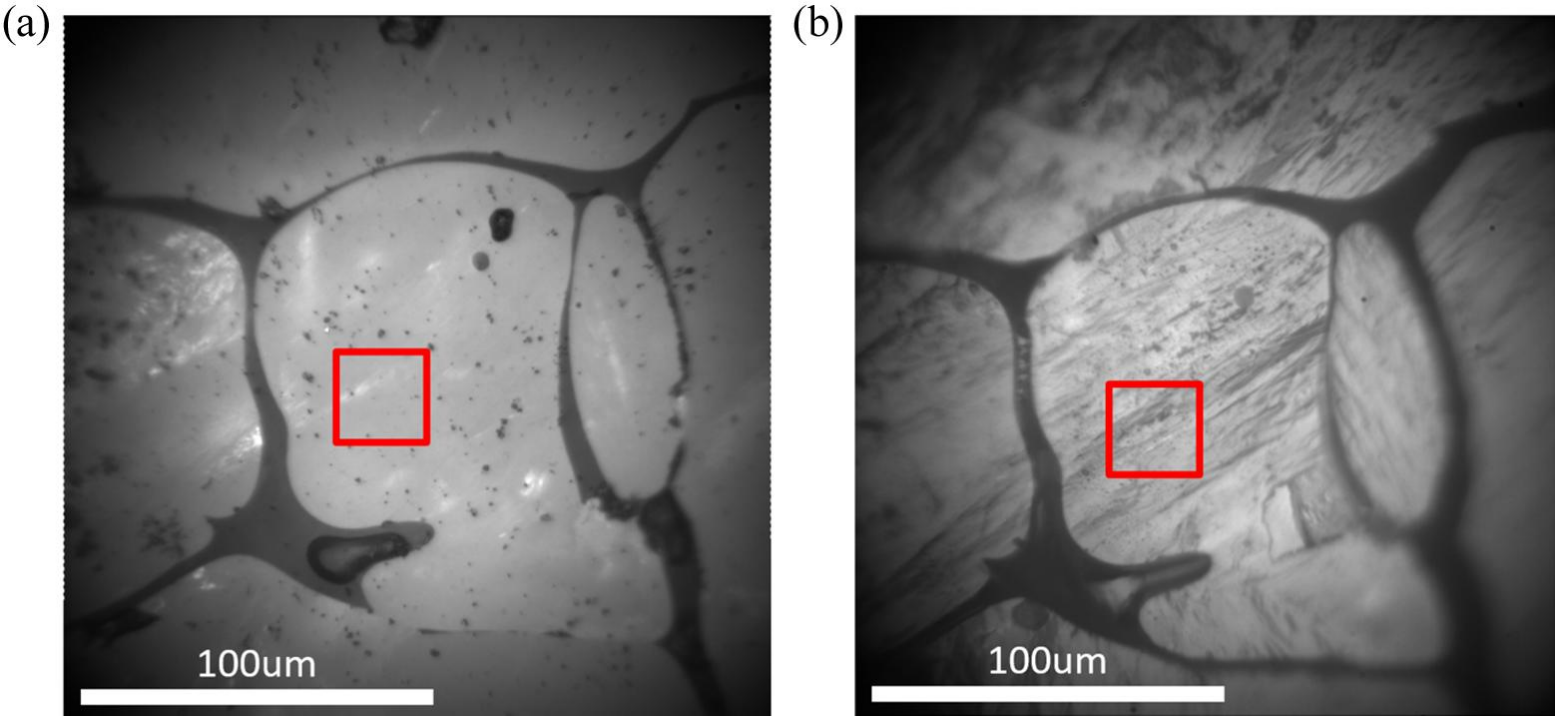
Optical images of the specimen obtained with the microscope of the beamline. The red square shows the ROI mapped by Laue diffraction. (*a*) The initial specimen at room temperature. (*b*) An image obtained at room temperature after one heating–cooling cycle (up to 1530 K). The dark grey is a glassy phase surrounding the light-grey zirconia.

Fig. 8 ▸ presents the optical image of the specimen surface in its initial stage at room temperature, and after heating to 1530 K and cooling back to room temperature. The red box on the figure represents the ROI (size: 30 × 30 µm) that was scanned with a grid of 60 × 60 Laue images, *i.e.* with an in-plane spatial resolution of 500 nm. To align at high temperature the incoming X-ray beam with the ROI, in the absence of an optical microscope, we took advantage of the presence of a silicate glassy phase (volume fraction of ∼5%), which appears in dark grey in Fig. 8 ▸. A fluorescence scan yields the location of this glassy phase, enabling us at each temperature to determine the position of the ROI relative to the glassy phase (micrometric resolution). As a result of the phase transformation, microstructural changes are displayed in the two optical-microscope images shown in Fig. 8 ▸.

**Figure 9:**
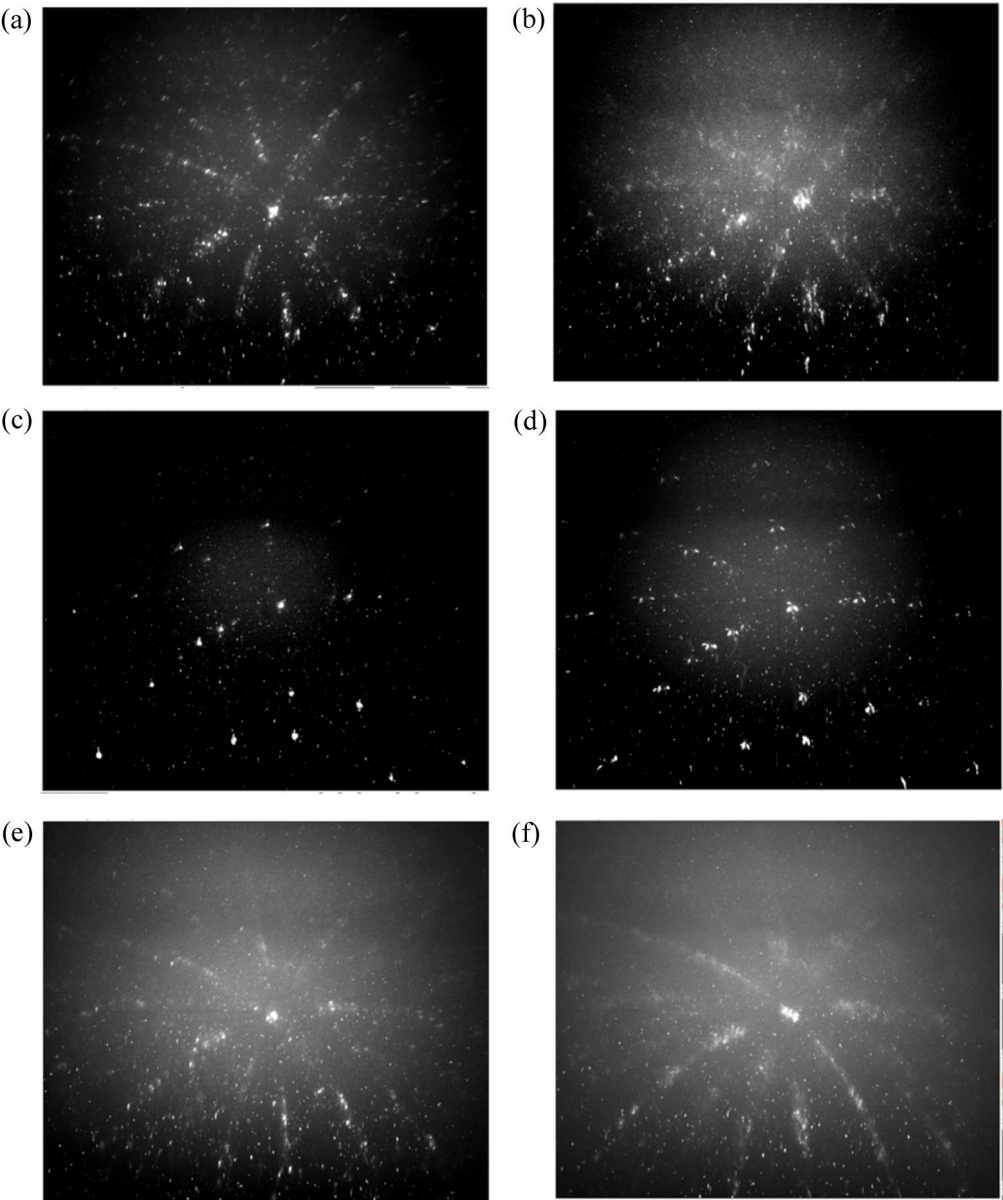
Laue diffraction patterns of zirconia at different temperatures during heating, (*a*)–(*c*), and then cooling, (*c*)–(*f*). (*a*) The initial specimen at room temperature, (*b*) 600 K, (*c*) 1530 K, (*d*) 1184 K, (*e*) 1063 K and (*f*) 727 K.

Fig. 9 ▸ presents a short selection of Laue images from the same location on the specimen (within a few micrometres resolution) at different temperatures. Fig. 9 ▸(*a*) shows Laue patterns of monoclinic ZrO_2_ at room temperature, before the thermal loading. A crystal structure can be recognized in this Laue pattern, although the image is crowded with many (thousands) small spots. Such crowding of Laue peaks comes from the fact that the actual *m*-ZrO_2_ crystals are nanometric in size (Guinebretière *et al.*, 2022 ▸), *i.e.* several orders of magnitude smaller than the gauge volume size (at 17.5 keV, the penetration depth of X-rays into zirconia is ∼150 µm), and therefore a single Laue image gathers diffraction spots from a very large number of individual crystals. As discussed above, the stress level in these nanometric crystals can be huge, in the gigapascal range, with huge stress gradients too (Ors *et al.*, 2021 ▸). On top of that, it has been shown by Humbert *et al.* (2010 ▸) that each ZrO_2_ region observed in Fig. 8 ▸, surrounded by the glassy phase, originates from a single cubic crystal above 2575 K, and therefore the investigated ROI (or the gauge volume) may contain up to 24 variants of the monoclinic phase. These 24 *m* variants populate the recorded Laue image.

At temperatures higher than ∼1250 K, zirconia transforms into a tetragonal structure. This is very clearly shown in Fig. 9 ▸(*c*) (1530 K). Here, a Laue pattern similar to those obtained for single crystals is observed. The gain in symmetry is evident from the disappearance of the crowded pattern as in Fig. 9 ▸(*a*). The (up to) 24 monoclinic variants at room temperature lead to only three variants at 1530 K. In fact, a single cubic grain at very high temperature gives rise to only three tetragonal variants, and from those three, apparently only one lies within the X-ray beam here. The observed Laue pattern in Fig. 9 ▸(*c*) probably comprises many *t* crystals with almost identical crystallographic orientations. A similar feature has also been recently observed using high-temperature 3D reciprocal-space maps, recorded on much larger gauge volumes (sub-millimetre cubed), where the 24 111 or 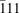 relatively spread RLNs of the monoclinic phase join together into three very well defined 111 RLNs of the tetragonal phase at 1423 K (Purushottam Raj Purohit *et al.*, 2022*b* ▸). To the best of our knowledge, Fig. 9 ▸(*c*) is the very first Laue microdiffraction image obtained above 1000 K. Challenges associated with stray diffraction spots from the beryllium dome in the high-temperature Laue experiments were overcome here through image-processing techniques. Alternatively, a dome constructed of PEEK could be utilized in future investigations to avoid this issue.

At 1184 K, the *t → m* transformation is engaged [Fig. 9 ▸(*d*)]. Some of the crystals under the tetragonal symmetry at 1423 K are now under the monoclinic form. A clear splitting of the tetragonal spots is observed. Cooling the specimen further to 727 K [Figs. 9 ▸(*e*) and 9 ▸(*f*)], we get a Laue pattern similar to the initial one, crowded with an even larger number of spots. However, one can recognize the main crystal orientations associated with the *m* variants, like for the initial room-temperature pattern.

**Figure 10:**
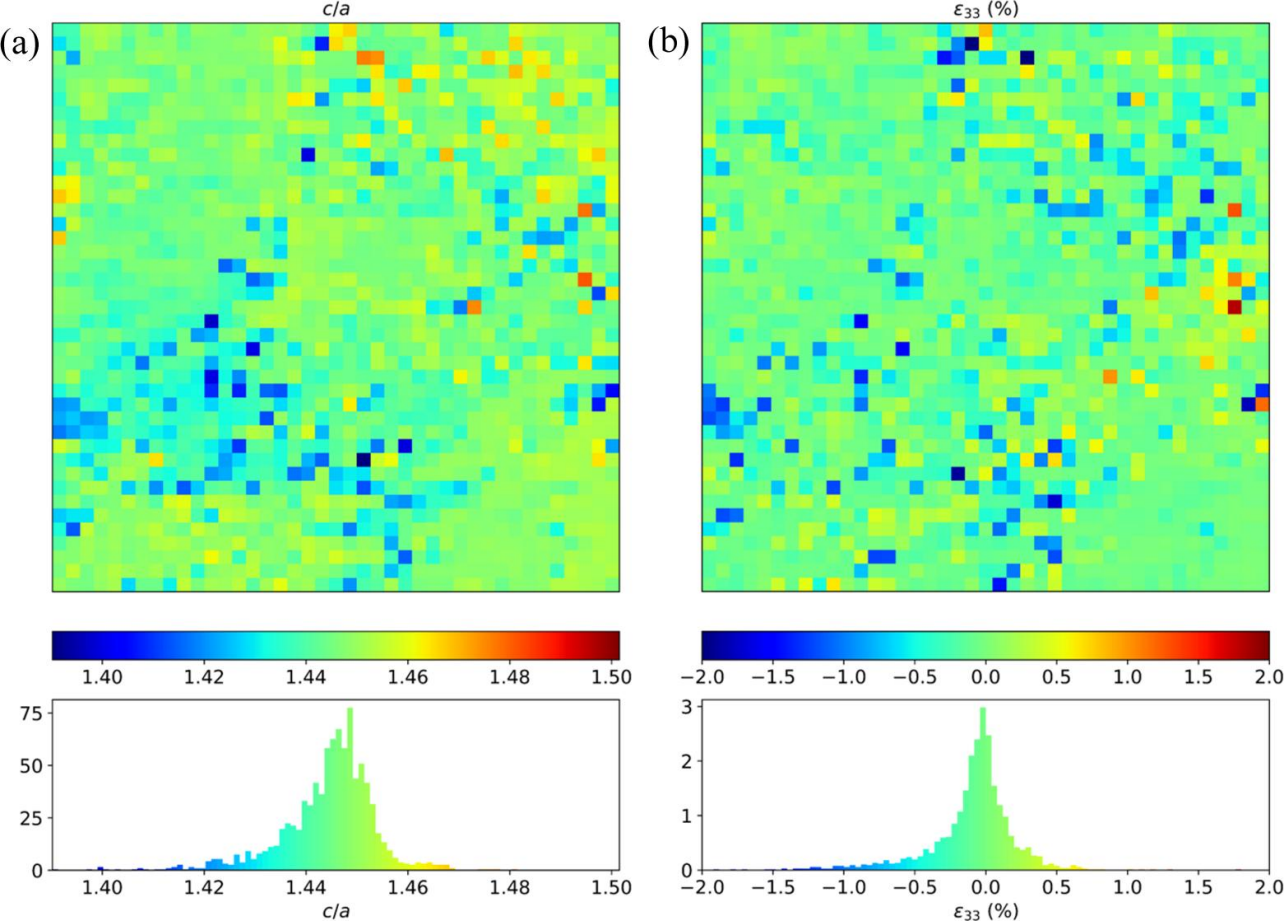
(*a*) A map of lattice-parameter ratio *c/a* of *t*-ZrO_2_ at 1530 K, and associated probability density. (*b*) Two-dimensional distribution and probability density of the deviatoric lattice strain along the *c* axis.

Alongside this, we also present in Fig. 10 ▸ the first-ever high-temperature cell-parameter fluctuation maps recorded with Laue microdiffraction, specifically at 1530 K for tetragonal zirconia. Concerning the processing of the Laue images, we have made use of the neural-network-based method denoted LaueNN (Purushottam Raj Purohit *et al.*, 2022*a* ▸), which allows very efficient indexing of all Laue spots, almost in real time during data acquisition. Once the Miller indexes of each Laue spot are found with the neural network, the lattice parameters of the deformed crystals can be determined using a least-squares minimization procedure (Chung & Ice, 1999 ▸). In Fig. 10 ▸, as an illustrative example, we show the 2D distribution of *c*/*a* cell parameters at 1530 K for the 30 × 30 µm scanned region indicated in Fig. 8 ▸ together with the deviatoric elastic strain along the *c* axis.

If one considers the average lattice parameter for this 2D map as a reference lattice parameter (this average value of *c*/*a* might differ from the stress-free lattice parameter due to residual stresses), one can compute the distribution of deviatoric strain along the *c* axis, denoted as ε_33_ in Fig. 10 ▸(*b*). A Gaussian-like distribution is obtained for this strain component, although other components (not shown here) do not necessarily follow a symmetrical distribution. Here, the standard deviation of ε_33_ is ∼1 × 10^−3^, and a significant part of the ROI encompasses deviatoric elastic strain up to 5 × 10^−3^. This corresponds to residual stress fluctuations (deviatoric axial stress along *c*) in the sub-gigapascal or gigapascal range. Such an estimation matches well with our recent full-field polycrystal computation accounting for the Bain strain associated with the *c* → *t* phase transition and the anisotropic thermal expansion of the *t* phase during the cooling to 1530 K (to be published).

## 5. Concluding remarks

This work constitutes, to the best of our knowledge, the first Laue microdiffraction measurements at very high temperatures (above 1500 K), using a new dedicated furnace available at beamline BM32, ESRF. We have introduced a calibration method for the experimental setup to offset the limitations imposed by the dome covering the specimen, which otherwise hinders the use of optical microscopy for precise alignment. This calibration approach has proven effective, substantiated by both synthetic and actual experimental data, enabling us to accurately gauge the evolution of lattice parameters at elevated temperatures.

We have also demonstrated the feasibility of employing a sapphire single crystal as a temperature sensor for the sample. Our findings reveal that the discrepancy between the set-point and actual sample temperatures remains minimal at higher temperatures, of the order of several tens of degrees. This is a crucial point for understanding material transformations at high temperatures.

As an illustrative example, we have shown the very first data obtained on a pure zirconia polycrystalline specimen undergoing a phase transition from monoclinic to tetragonal states upon heating. The acquired Laue patterns are fully consistent with previous analyses of the splitting of the 111 reciprocal-space node (Purushottam Raj Purohit *et al.*, 2022*b* ▸), where the specimen passes from a highly stressed microstructure comprising 24 variants to a simpler one with only three variants, although we show here that the distribution of the stress level in the tetragonal phase still lies in the gigapascal range (in agreement with forthcoming full-field computation results).

This high-temperature Laue setup opens a wide range of new opportunities in the evaluation of crystalline material transformations at very high temperatures. These include real-time mapping of phase-transition microstructures and the corresponding local stress and strain fields.
